# Supplementary material for: Cryo EM structures map a post vaccination polyclonal antibody response to canine parvovirus
Source: Commun Biol. 2023 Sep 19;6:955. doi: 10.1038/s42003-023-05319-7 (PMC10509169; doi:10.1038/s42003-023-05319-7)
Supplement: Supplementary file 2 — Supplementary Material [file 42003_2023_5319_MOESM2_ESM.pdf]

SUPPLEMENTAL MATERIAL

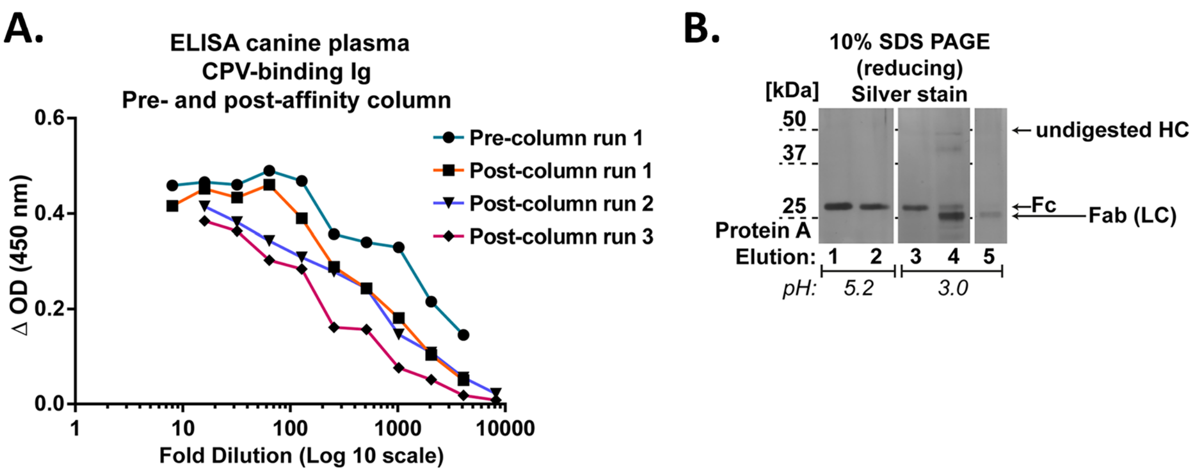

Supplemental Figure 1. CPV-binding Fab isolation by affinity chromatography. (A) Reduction of CPV-binding Ig in the canine plasma sample after each run on the affinity column. (B) 10% SDS PAGE and silver stain of eluted fractions from Protein A purification of papain-digested Ig. Fc fragments eluted at intermediate pH 5.2, Fab at low pH 3.0. Fab containing eluted fractions 4 and 5 were collected (affinity purified Fab) and were analyzed by cryo EM; (A) n = 1 experiment.

A.

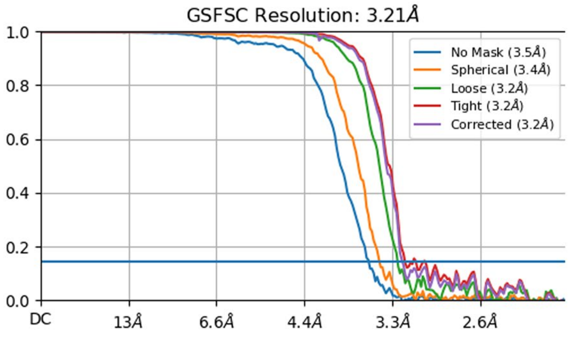

B.

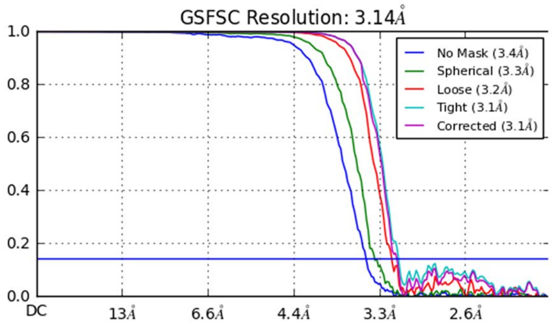

Supplemental Figure 2. Icosahedral Reconstruction FSC Curves. (A) Affinity purified polyclonal Fab icosahedral reconstruction FSC curve with cutoff 0.143. (B) Affinity purified polyclonal Fab icosahedral reconstruction FSC curve with cutoff 0.143.

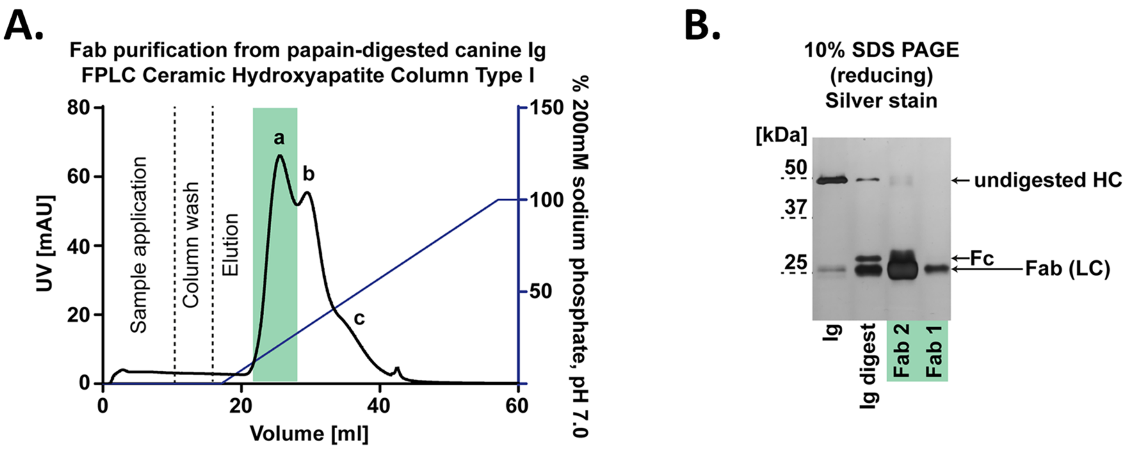

Supplemental Figure 3. Verification of canine CPV-binding Fab isolation by affinity chromatography. (A) Chromatogram of papain digested canine Ig separated by mixed-mode chromatography; peak a was caused by Fab that were collected for cryo EM, peak b was caused by Fc fragments, and peak c by undigested Ig. (B) 10% SDS PAGE and silver stain of canine Ig, papain-digested Ig and purified polyclonal Fab 1 and 2 were combined before incubating with CPV capsids.

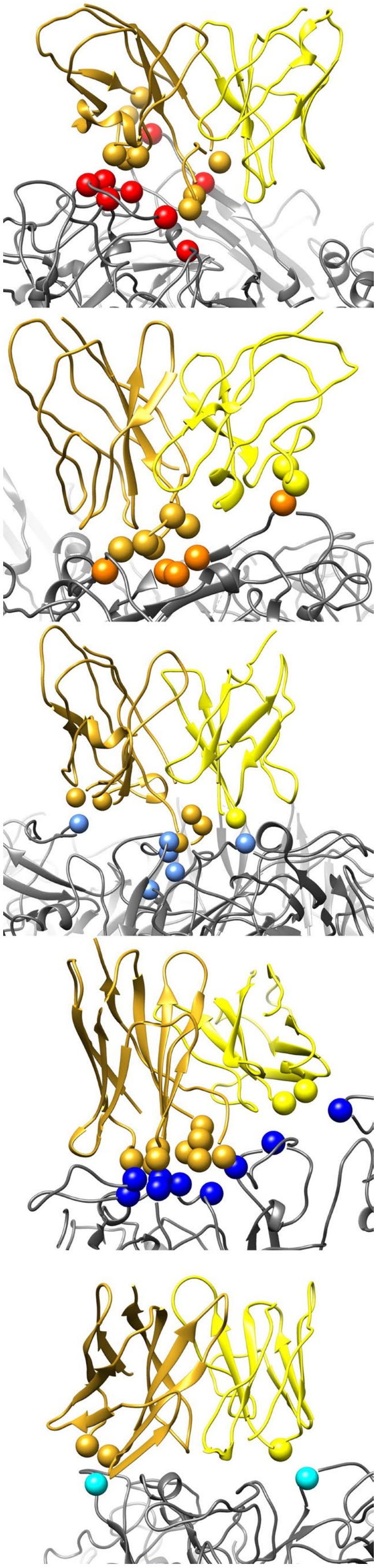

Supplemental Figure 4. Interface of Polyclonal-Virus Complexes.

The binding interface of the polyclonal Fabs to the virus is shown. Capsid protein VP2 is gray, Fab heavy chain gold and Fab light chain yellow. Contacts between the Fab and the virus are shown as spheres, with the paratope matching the Fab chain color and epitope matching the consensus color scheme in Figure 7. In descending order: A site Fab affinity purified (red) and total-Fab (orange); B site Fab affinity purified (cornflower blue), total-Fab B1 and B2 (blue and cyan).

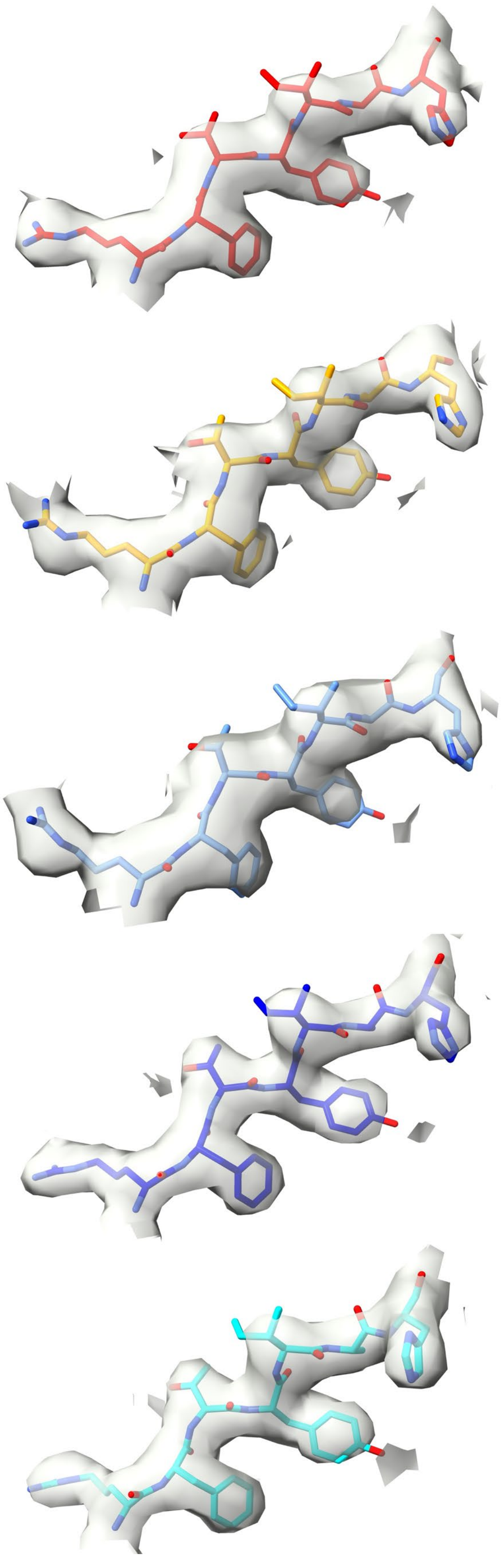

Supplemental Figure 5. Example Fit of Models to the Map.

Examples of the fit of the model to the map are shown with the carbons colored: A site Fab affinity purified (red) and total-Fab (orange); B site Fab affinity purified (cornflower blue), total-Fab B1 and B2 (blue and cyan). Other atoms are colored by heteroatom. The region featured comprises VP2 residues 397 to 403.

Supplemental Table 1. Vaccination schedule for male dog (Beagle) (Sample 1).

Age [days]	Type	Name
22	Bordetella Bronchiseptica, Parainfluenza, Canine Adenovirus Type 2	Intra-Trac 3
**29**	**Canine Parvovirus**	NeoPar
**36**	**Canine Parvovirus**	NeoPar
43	Bordetella Bronchiseptica Bacterin	Bronchicine CAe
**51**	**Canine Parvovirus**	NeoPar
58	*Blood collection*	

Supplemental Table 2. Cryo-EM data collection

Data Collection and Processing	Affinity Purified Fab- CPV	Total-Fab Purified – CPV
Magnification	59,000	59,000
Voltage (kV)	300	300
Electron Exposure (e-/Å^2)	45	45
Defocus Range (um)	0.5-3.0	0.5-3.0
Pixel Size (Å)	1.1	1.1
Symmetry Imposed	I1	I1
Micrographs Collected	6,907	8,124
Initial Particle Number	193,445	343,104
Final Particle Number	184,559	322,846
Map Resolution (Å)	3.21	3.14
FSC Threshold	0.143	0.143
Subparticles per Particle- A Site	60	60
Final Subparticle Number- A Site	11,073,540	19,370,760
Subparticles per Particle- B Site	60	60
Final Subparticle Number- B Site	11,073,540	19,370,760

Supplemental Table 3. Vaccination schedule for male dog (Beagle) (Sample 2).

Age [days]	Type	Name
59	Bordetella Bronchiseptica, Parainfluenza, Canine Adenovirus Type 2	Intra-Trac 3
**59**	**Canine Parvovirus**	NeoPar
**65**	**Canine Parvovirus**	NeoPar
65	Canine Papilloma (Types 1&2)	Papilloma
**71**	Distemper, Canine Adenovirus Type 2, Parainfluenza, **Canine Parvovirus**, Leptospira Bacterin	Nobivac 1-DAPPv+L4
**72**	**Canine Parvovirus**	NeoPar
77	Bordetella Bronchiseptica Bacterin	Bronchicine CAe
79	Canine Papilloma (Types 1&2)	Papilloma
86	*Blood collection*	

Supplemental Table 4. Refinement Statistics Affinity Purified Dataset.

Refinement	Affinity Purified A Site (Red)	Affinity Purified B Site (Cornflower)
Model composition		
Non-hydrogen atoms	31056	32765
Protein Residues	3762	3976
R.m.s. Deviations		
Bond Length (Å)	0.007	0.007
Bond Angles (°)	1.104	0.920
Validation		
MolProbity Score	1.43	0.98
Clash Score	3.73	0.73
Rotamer Outliers (%)	0.36	0.66
Ramachandran Plot		
Favored (%)	96.09	96.34
Outliers (%)	0.03	0.00

Supplemental Table 5. Refinement Statistics Total-Fab Dataset.

Refinement	**Total-Fab** A Site (Orange)	**Total-Fab** B Site 1 (Blue)	**Total-Fab** B Site 2 (Cyan)
Model composition			
Non-hydrogen atoms	25826	33004	32548
Protein Residues	3092	4003	3957
R.m.s. Deviations			
Bond Length (Å)	0.008	0.006	0.009
Bond Angles (°)	0.965	0.989	0.923
Validation			
MolProbity Score	1.02	1.23	0.93
Clash Score	0.78	2.87	0.87
Rotamer Outliers (%)	0.99	0.26	1.24
Ramachandran Plot			
Favored (%)	95.99	97.18	97.54
Outliers (%)	0.00	0.00	0.03
