# Supplementary material for: Cryo EM structures map a post vaccination polyclonal antibody response to canine parvovirus
Source: Commun Biol. 2023 Sep 19;6:955. doi: 10.1038/s42003-023-05319-7 (PMC10509169; doi:10.1038/s42003-023-05319-7)
Supplement: Supplementary file 3 — Description of Additional Supplementary Files [file 42003_2023_5319_MOESM3_ESM.pdf]

Description of Additional Supplementary Files

File name: Supplementary Data 1

Description: Source data for the graphs found in the supplementary materials.
