# Supplementary material for: Cryo EM structures map a post vaccination polyclonal antibody response to canine parvovirus
Source: Commun Biol. 2023 Sep 19;6:955. doi: 10.1038/s42003-023-05319-7 (PMC10509169; doi:10.1038/s42003-023-05319-7)
Supplement: Supplementary file 4 — Reporting Summary [file 42003_2023_5319_MOESM4_ESM.pdf]

nature portfolio

Corresponding author(s): Susan Hafenstein

Last updated by author(s): 2023-08022

Reporting Summary

Nature Portfolio wishes to improve the reproducibility of the work that we publish. This form provides structure for consistency and transparency in reporting. For further information on Nature Portfolio policies, see our Editorial Policies and the Editorial Policy Checklist.

Statistics

For all statistical analyses, confirm that the following items are present in the figure legend, table legend, main text, or Methods section.

n/a	Confirmed	
☒	☐	The exact sample size (*n*) for each experimental group/condition, given as a discrete number and unit of measurement
☒	☐	A statement on whether measurements were taken from distinct samples or whether the same sample was measured repeatedly
☒	☐	The statistical test(s) used AND whether they are one- or two-sided<br>*Only common tests should be described solely by name; describe more complex techniques in the Methods section.*
☒	☐	A description of all covariates tested
☒	☐	A description of any assumptions or corrections, such as tests of normality and adjustment for multiple comparisons
☒	☐	A full description of the statistical parameters including central tendency (e.g. means) or other basic estimates (e.g. regression coefficient) AND variation (e.g. standard deviation) or associated estimates of uncertainty (e.g. confidence intervals)
☒	☐	For null hypothesis testing, the test statistic (e.g. *F*, *t*, *r*) with confidence intervals, effect sizes, degrees of freedom and *P* value noted<br>*Give P values as exact values whenever suitable.*
☒	☐	For Bayesian analysis, information on the choice of priors and Markov chain Monte Carlo settings
☒	☐	For hierarchical and complex designs, identification of the appropriate level for tests and full reporting of outcomes
☒	☐	Estimates of effect sizes (e.g. Cohen's *d*, Pearson's *r*), indicating how they were calculated

Our web collection on statistics for biologists contains articles on many of the points above.

Software and code

Policy information about availability of computer code

Data collection	EPU (E pluribus unum) 2.6.1-3.13.0 commercial software on Krios microscope used to collect data.
Data analysis	CryoSPARC 3.3.2, Relion 3.1, ISECC (icosahedral subparticle extraction and correlative classification), and DeepEMhancer 23.1.12 are all freeware used to reconstruct the cryo EM data. Free software for model building includes Coot 0.9.8.5, ISOLDE 4.1.11.6, and Phenix 1.20.1.

For manuscripts utilizing custom algorithms or software that are central to the research but not yet described in published literature, software must be made available to editors and reviewers. We strongly encourage code deposition in a community repository (e.g. GitHub). See the Nature Portfolio guidelines for submitting code & software for further information.

Data

Policy information about availability of data

All manuscripts must include a data availability statement. This statement should provide the following information, where applicable:

- Accession codes, unique identifiers, or web links for publicly available datasets
- A description of any restrictions on data availability
- For clinical datasets or third party data, please ensure that the statement adheres to our policy

The cryo EM maps and protein structures of the refined polyclonal fab CPV complexes, are deposited in the EM data bank (www.emdatabank.org/) and in the PDB (https://www.rcsb.org). For the affinity purified dataset the A site subparticle with Fab bound (red colored Fab in Figure 7) (PDB: 7UTP, EMD- 26786) and the B site subparticle with Fab bound (cornflower blue colored Fab in Figure 7) (PDB: (7UTR, EMD-26787) were deposited. For the total-Fab dataset the A site subparticle with

Fab bound (orange colored Fab in Figure 7) (PDB: 7UTS, EMD-26788), the B site subparticle Fab B1 with Fab bound (blue colored Fab in Figure 7) (PDB: 7UTU, EMD-26789), and the B site subparticle Fab B2 with Fab bound (cyan colored Fab in Figure 7) (PDB: 7UTV, EMD-26790) were deposited. Note: the corresponding whole particle icosahedrally averaged sharpened and unsharpened maps for the respective datasets are available as additional maps under each corresponding accession ID.

Research involving human participants, their data, or biological material

Policy information about studies with human participants or human data. See also policy information about sex, gender (identity/presentation), and sexual orientation and race, ethnicity and racism.

Reporting on sex and gender	na
Reporting on race, ethnicity, or other socially relevant groupings	na
Population characteristics	na
Recruitment	na
Ethics oversight	na

Note that full information on the approval of the study protocol must also be provided in the manuscript.

Field-specific reporting

Please select the one below that is the best fit for your research. If you are not sure, read the appropriate sections before making your selection.

☒ Life sciences ☐ Behavioural & social sciences ☐ Ecological, evolutionary & environmental sciences

For a reference copy of the document with all sections, see nature.com/documents/nr-reporting-summary-flat.pdf

Life sciences study design

All studies must disclose on these points even when the disclosure is negative.

Sample size	for cryo EM single particle reconstruction, 184000-323000 particles per dataset
Data exclusions	duplicate particles are excluded
Replication	thousands of projection images are averaged together
Randomization	thousands of projection images are in myriad orientations
Blinding	no blinding operations are done for single particle cryo EM reconstruction

Reporting for specific materials, systems and methods

We require information from authors about some types of materials, experimental systems and methods used in many studies. Here, indicate whether each material, system or method listed is relevant to your study. If you are not sure if a list item applies to your research, read the appropriate section before selecting a response.

Materials & experimental systems

n/a	Involved in the study	
☐	☒	Antibodies
☐	☒	Eukaryotic cell lines
☒	☐	Palaeontology and archaeology
☐	☒	Animals and other organisms
☒	☐	Clinical data
☒	☐	Dual use research of concern
☒	☐	Plants

Methods

n/a	Involved in the study	
☒	☐	ChIP-seq
☒	☐	Flow cytometry
☒	☐	MRI-based neuroimaging

Antibodies

Antibodies used	Canine polyclonal serum, isolated direct from animal
Validation	The canine polyclonal antibodies being investigated are the experimental subject, and were obtained from dogs with known histories of vaccination - this is described in detail as part of the Materials and Methods, as well as in the Results.

Eukaryotic cell lines

Policy information about cell lines and Sex and Gender in Research

Cell line source(s)	Cell lines were only used in the preparation of parvovirus capsids. The cells used were the Crandell Reese Feline Kidney (CRFK) cell line, which was obtained from the American Type Culture Collection (ATCC).
Authentication	Were authenticated by the ATCC.
Mycoplasma contamination	Cells were tested and were free of mycoplasma.
Commonly misidentified lines (See ICLAC register)	Not applicable.

Animals and other research organisms

Policy information about studies involving animals; ARRIVE guidelines recommended for reporting animal research, and Sex and Gender in Research

Laboratory animals	The animals were dogs that were being raised in a breeding facility.
Wild animals	na
Reporting on sex	These studies do not consider sex as a variable, as only two animals were used in this study. In the future comparison of male and female dogs, as well as dogs of different ages would be included.
Field-collected samples	na
Ethics oversight	All procedures involving animals were approved by Cornell University Institutional Animal Care and Use Committee (IAUCUC) (Protocol ID: 2017-0085)

Note that full information on the approval of the study protocol must also be provided in the manuscript.
